# The usefulness of endoscopic ultrasound in the diagnosis of gallbladder lesions

**DOI:** 10.3389/fmed.2022.957557

**Published:** 2022-08-29

**Authors:** Takashi Tamura, Reiko Ashida, Masayuki Kitano

**Affiliations:** Second Department of Internal Medicine, Wakayama Medical University, Wakayama, Japan

**Keywords:** gallbladder tumor, endoscopic ultrasound (EUS), contrast-enhanced harmonic endoscopic ultrasonography (CH-EUS), endoscopic ultrasound-guided fine needle aspiration (EUS-FNA), gallbladder carcinoma (GBC)

## Abstract

Gallbladder tumors are neoplastic lesions; however, it can be difficult to distinguish between benign and malignant gall bladder tumors before surgery, although endoscopic ultrasound (EUS) is useful for differentiation. Fundamental B mode EUS (FB-EUS) and contrast-enhanced harmonic EUS (CH-EUS) are reported to be useful for the diagnosis of gallbladder tumor because they allow evaluation of polypoid lesion and gallbladder wall thickening. Scoring systems based on FB-EUS imaging are available for the diagnosis of malignant gallbladder polypoid lesions. The characteristic findings of malignant gallbladder polypoid lesions on CH-EUS include the presence of irregular intratumoral vessels and perfusion defects. The characteristic findings of malignant gallbladder wall thickening on FB-EUS include wall thickening >12 mm, hypoechoic internal echogenicity, inhomogeneous internal echo pattern, and disrupted wall layer, whereas CH-EUS findings include hypovascular enhancement and inhomogeneous contrast distribution pattern. In addition, FB-EUS and CH-EUS are useful for evaluating the stage of gallbladder carcinoma because they allow the evaluation of the depth of invasion of the gallbladder wall. It is usually difficult to obtain pathological evidence from gallbladder tumors before surgery and chemotherapy, even though the histological diagnosis is necessary for determining treatment policy. EUS-guided fine needle aspiration (EUS-FNA) is useful for obtaining pathological samples from gallbladder tumors before surgery and chemotherapy. The accuracy rate of EUS-FNA for gallbladder tumor is as high as 90%, but complications such as bile leakage and needle track seeding can be a problem, although it was reported that contrast-enhanced harmonic imaging is useful for avoiding them.

## Introduction

Endoscopic ultrasound (EUS) is an essential examination in the diagnosis of diseases of the pancreaticobiliary system. EUS is reported to be useful for diagnosing benign and malignant gallbladder lesions and determining the invasion depth of gallbladder cancer (1). Recently, contrast-enhanced harmonic EUS (CH-EUS), which allows evaluation of blood flow, has enabled a more accurate diagnosis of gallbladder tumors than fundamental B mode EUS (FB-EUS) (2, 3). EUS also plays a major role in the histological diagnosis of gallbladder lesions, with EUS-guided fine needle aspiration (EUS-FNA) being reported to be useful for obtaining tissue samples of gallbladder tumor for pathological evaluation (4, 5). This review describes the usefulness of EUS in the diagnosis and analysis of gallbladder lesions.

## A. EUS imaging

### Methods of EUS imaging

#### Fundamental B mode EUS

Transabdominal ultrasound (TUS) is a minimally invasive examination. Therefore, TUS is a useful modality for screening for gallbladder lesions. However, it is sometimes difficult to differentiate malignant neoplasms from benign neoplasms on TUS alone because gallbladder cancer can take many different forms. FB-EUS can obtain higher resolution images than TUS in gallbladder lesions because the transducer can be positioned closer to the gallbladder lesion. The high-resolution image of FB-EUS makes it possible to evaluate a detailed view of the changes in the layered structure of the gallbladder wall and the internal echoes of the tumor, which are difficult to evaluate with TUS. The gallbladder can generally be observed when the EUS transducer is guided to the duodenal bulb or gastric antrum, although it may be difficult to observe some cases on FB-EUS because the location of the gallbladder differs widely from person to person.

#### Contrast harmonic mode

The CH-EUS is reportedly useful for evaluating the vascularity of gallbladder lesions through the use of second-generation ultrasound contrast agents in the diagnosis of gallbladder lesions (3, 6–8). Three types of contrast agents are currently available, Sono Vue (sulfur hexafluoride microbubbles; Bracco, Italy), Definity (octafluoropropane microbubbles; Bristol-Myers Squibb Medical Imaging, USA), and Sonazoid (perfluorobutane microbubbles; GE Healthcare, USA; Daiichi Sankyo, Japan). Sonazoid uses bubbles of perfluorobutane covered with a lipid membrane. The contrast agent has a very low molecular weight, and therefore, a low risk of allergic reaction. It can be used for patients with liver dysfunction and renal dysfunction because it is excreted through respiration. The contrast agent is mixed with distilled water for use as a white liquid. Sonazoid is intravenously injected at 15 μg/kg.

First, the gallbladder lesion is depicted on FB-EUS, and then the screen is changed to dual screen format with FB-EUS mode and CH-EUS mode imaging. The focus point should be set at the bottom. The penetration of the ultrasound beam on CH-EUS is inferior to that on FB-EUS mode imaging, and therefore, the target lesion should be imaged as closely as possible before CH-EUS is performed. Sonazoid is injected intravenously, followed by 10 ml of physiological saline. Gallbladder lesions will typically show enhancement 10–30 s after ultrasound contrast agent injection, and vascular and enhancement patterns are assessed in real time by examining continuous imaging over 0–15 (vascular imaging) and 40–60 s (perfusion images) post-injection.

### Diagnosis of gallbladder polypoid lesions

#### Fundamental B mode

Gallbladder polyps are often asymptomatic. Therefore, they are discovered incidentally during comprehensive health examinations or examinations for other medical purposes. Gallbladder polypoid lesions are classified as neoplastic or non-neoplastic, and epithelial or non-epithelial according to their microscopic structure and invasive characteristics (2). Polypoid lesions of the gallbladder should be differentiated at early stage gallbladder carcinoma from other benign lesions such as cholesterol polyp, hyperplastic polyp, metaplastic polyp, and inflammatory polyp (9, 10). In small (<2 cm) polypoid lesions of the gallbladder, the utility of EUS has been demonstrated with diagnostic sensitivity and specificity reported to be higher than on TUS (1, 11, 12).

The most common non-neoplastic gallbladder polys are cholesterol polyps. On FB-EUS imaging, cholesterol polyps present as homogeneous hyperechoic pedunculated multiple lesions smaller than 4 mm (13–15). In polyps of more than 10 mm, it can sometimes be difficult to distinguish cholesterol polyps from adenoma and gallbladder cancer because of epithelial hyperplastic changes being reflected as lobulation and reduced internal echo. In addition, it is reported that neoplastic lesions have the presence of hypoechoic foci.

In gallbladder polypoid lesions, tumor diameter is also an important finding, and a large size is independently associated with neoplastic polyps (*p* < 0.05). For polyps greater than 14 mm in diameter, the sensitivity for differentiating neoplastic from non-neoplastic polyps has been reported as 92.3% (16). On FB-EUS imaging, pedunculated polypoid lesions with a granular contour and spotty internal echo pattern indicate benign pathology, whereas the absence of these findings should raise suspicion of the neoplastic lesion (11, 17). In Joint European guidelines, cholecystectomy is recommended for polyps of more than 10 mm, although 5% of polyps <10 mm are also reported to be malignant (13, 18) (Table 1).

**Table 1:** Characteristic findings of gallbladder polyps in FB-EUS.

	**EUS findings**	**Non-neoplastic**	**Neoplastic**
Gallbladder polyp	Tumor diameter	Small	Greater than 14 mm
	Internal echo	Homogeneous	Heterogeneous
	Hyperechoic spot	Presence	Absence

FB-EUS, fundamental B-mode endoscopic ultrasound.

**Figure 1:**
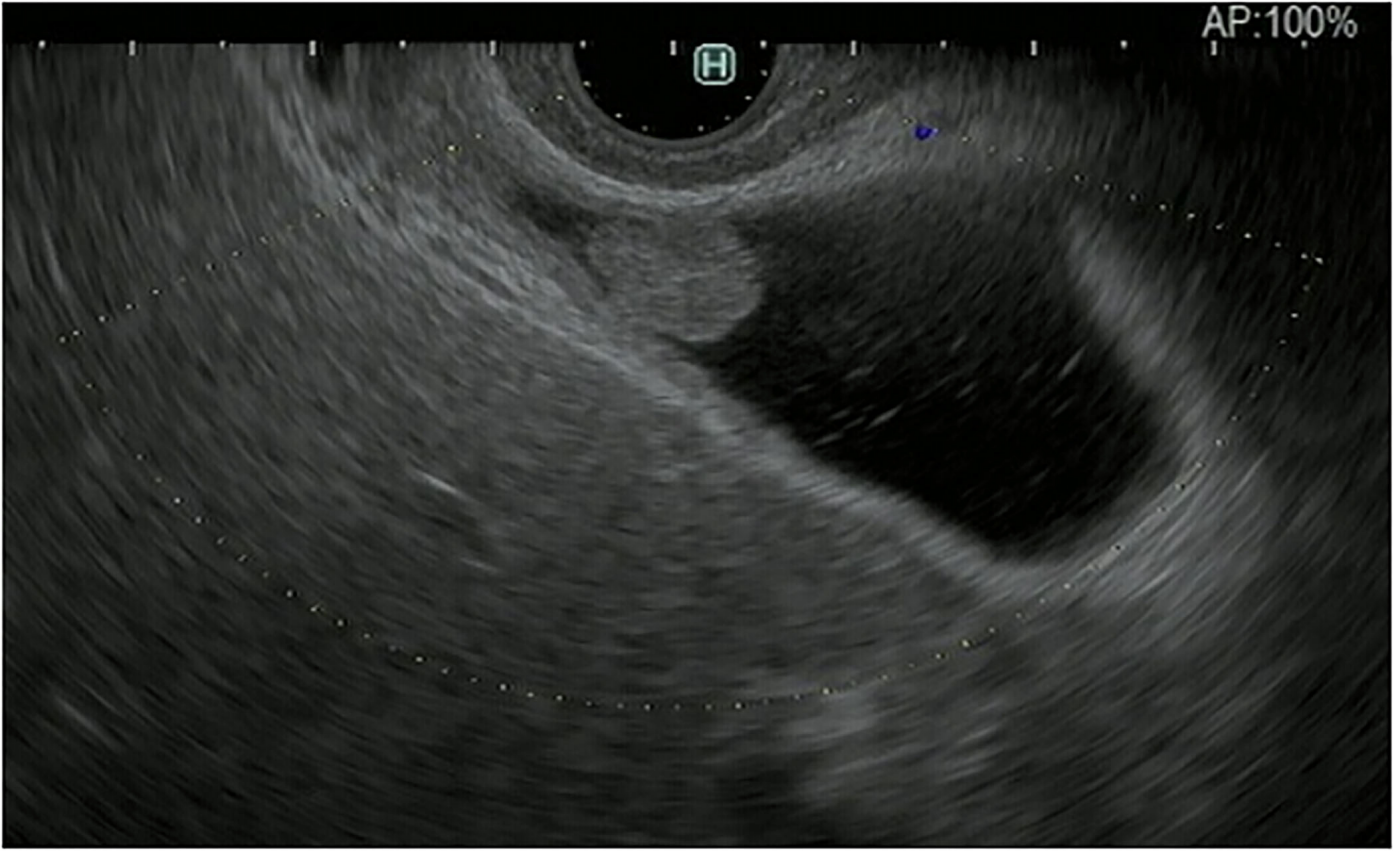
Fundamental B-mode EUS for gallbladder polypoid lesions. Gallbladder adenoma: iso-echoic homogenous pedunculated mass lesion.

**Figure 2:**
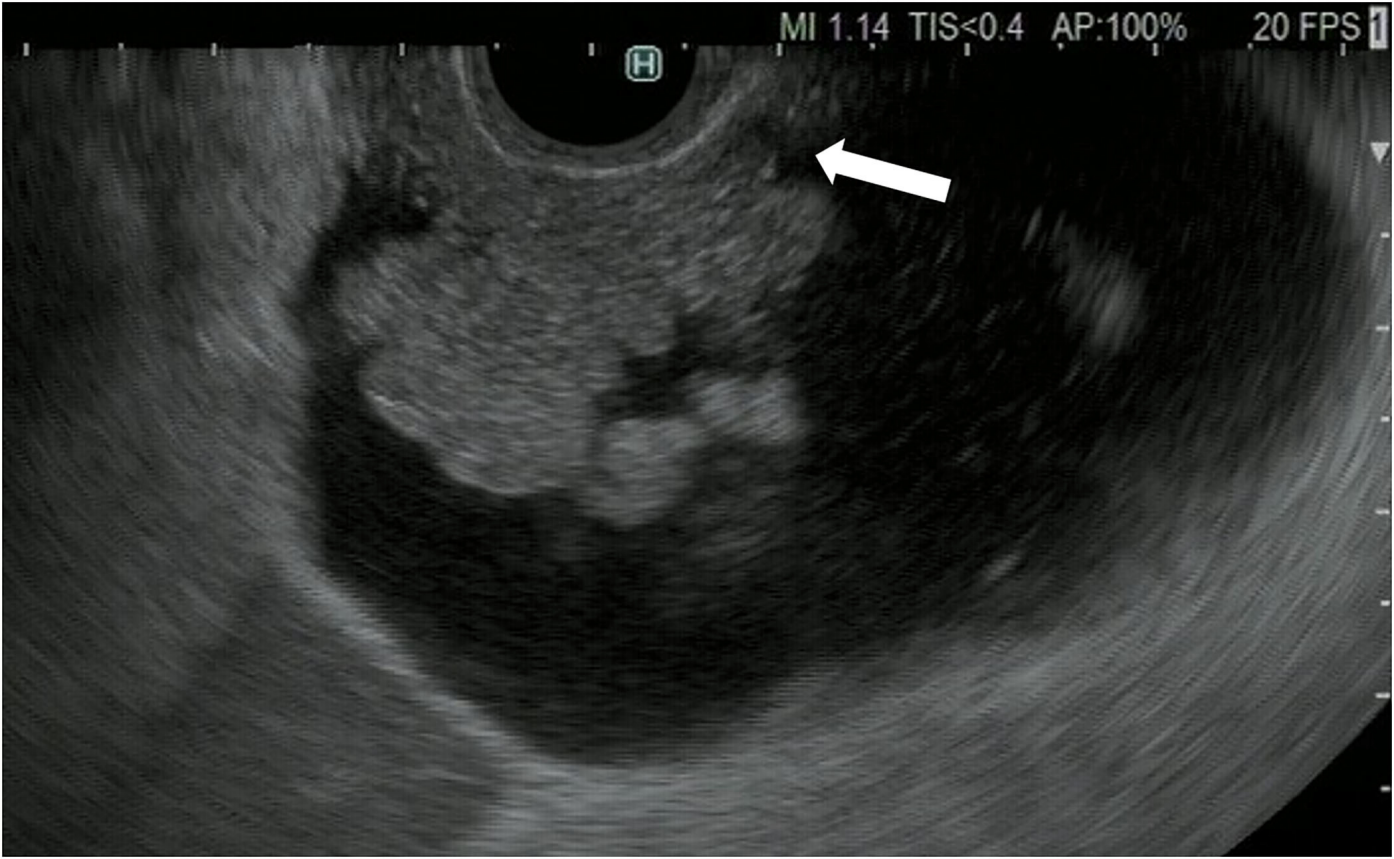
Fundamental B-mode EUS for gallbladder polypoid lesions. Gallbladder adenocarcinoma: internal hypoechoic heterogenous papillary elevated mass lesion (arrow).

There are two FB-EUS scoring systems for differentiating between non-neoplastic and neoplastic polyps. One for the differential diagnosis of gallbladder polyps from 5 to 15 mm in size is based on the layer structure, echo patterns, polyp margin, presence of a stalk, and number of polyps (19). The sensitivity and specificity of this scoring system were 81 and 86%, respectively, in the differential diagnosis of non-neoplastic and neoplastic polyps. The other FB-EUS scoring system is based on maximum diameter, internal echo pattern, and hyperechoic spotting. This scoring system showed sensitivity and specificity of 77.8 and 82.7% at the cut-off score of 12, respectively, in the differential diagnosis of non-neoplastic and neoplastic polyps (20) (Table 2). However, some studies have reported a limitation of FB-EUS for differentiating between non-neoplastic and neoplastic polyps of <1 cm (12) (Figures 1, 2).

**Table 2:** FB-EUS scoring system by Sadamoto et al.

**Variables**		**Score**
**Maximum diameter (mm)**		**Maximum diameter (mm)**
Internal echo	Heterogeneous	+4
	Homogeneous	0
Hyperechoic spot	Presence	−5
	Absence	0
Total score		

FB-EUS, fundamental B-mode endoscopic ultrasound.

#### Contrast harmonic mode

**Figure 3:**
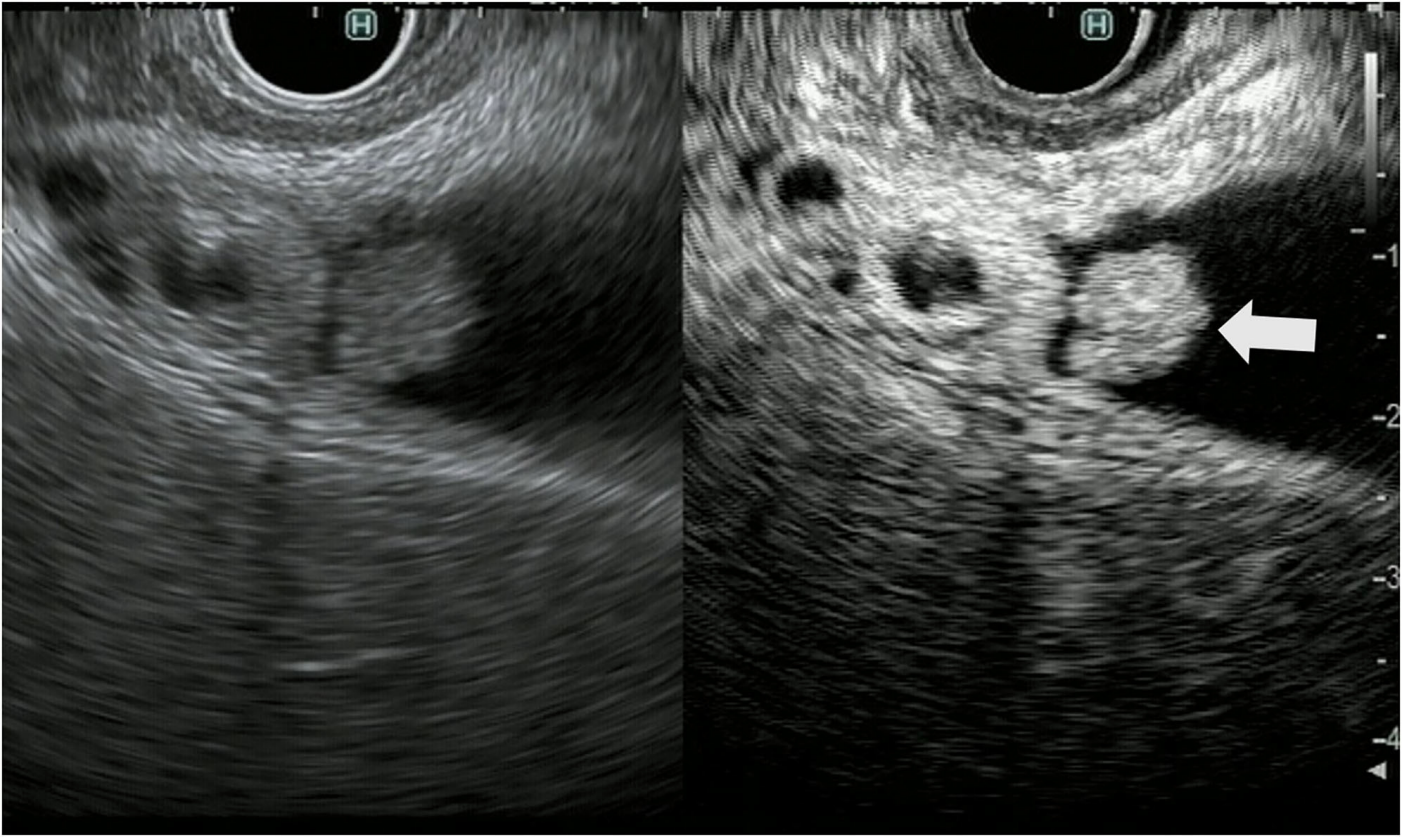
Contrast-enhanced harmonic endoscopic ultrasound (EUS) for gallbladder polypoid lesions. Gallbladder adenoma: The mass is a polypoid lesion with homogeneous hyperenhancement (arrow).

**Figure 4:**
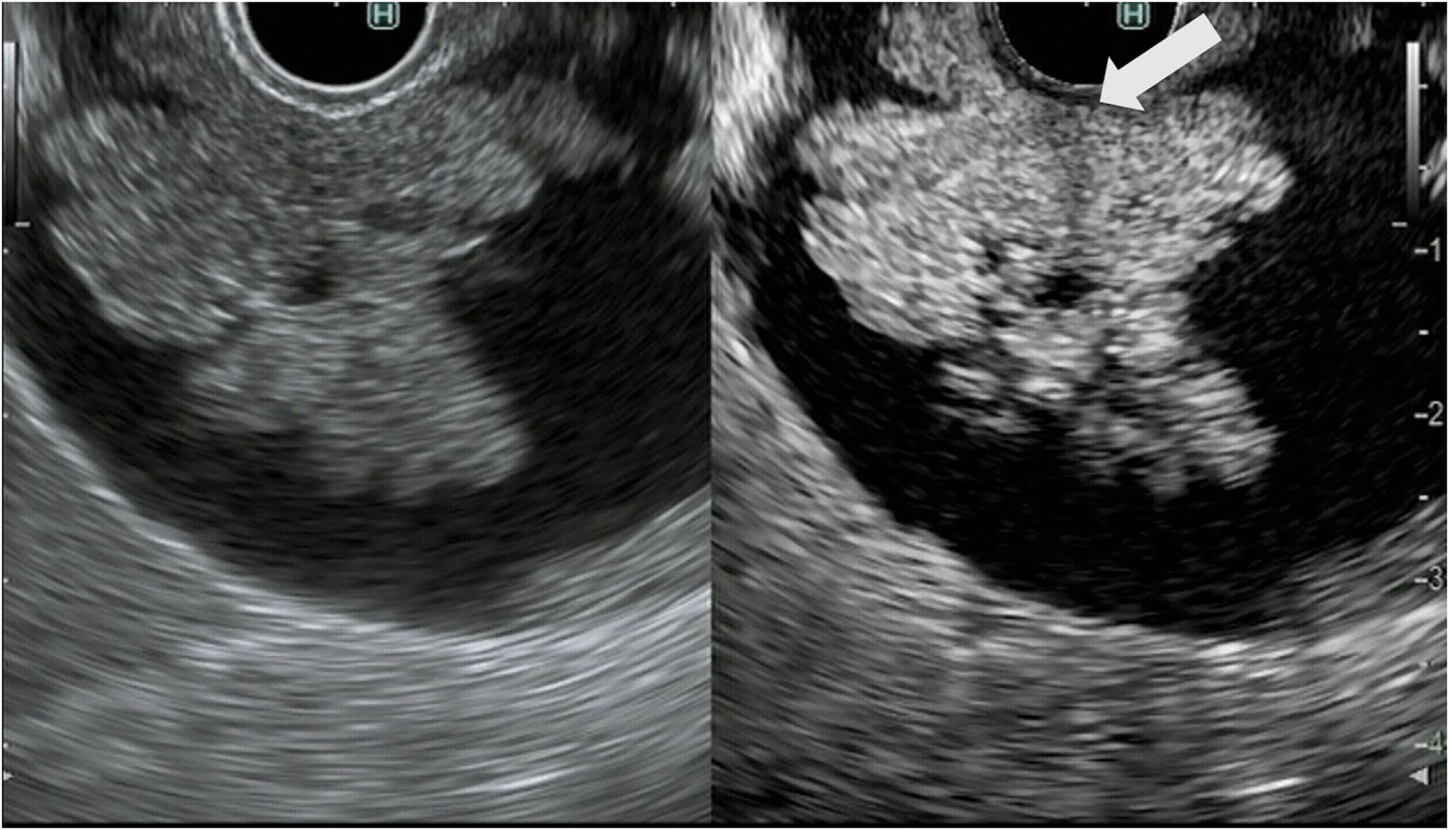
Contrast-enhanced harmonic endoscopic ultrasound (EUS) for gallbladder polypoid lesions. Gallbladder adenocarcinoma: The polypoid lesion has a perfusion defect area (arrow).

In gallbladder polyps with a maximum diameter of at least 10 mm, the presence of irregular intratumoral vessels and a perfusion defect are reportedly the characteristic findings of gallbladder cancer on CH-EUS imaging (Table 3). The sensitivity and specificity for the diagnosis of malignant polyps with irregular intratumoral vessel patterns on CH-EUS imaging are 90.3 and 96.6%, respectively (6), while the sensitivity and specificity for those showing the presence of perfusion defects are 90.3 and 94.9%, respectively. In the diagnosis of malignant polyps, the sensitivity and specificity of CH-EUS are superior to those of FB-EUS (CH-EUS *vs*. FB-EUS, 93.5 and 93.2%, respectively, *vs*. 90.0 and 91.1%; Figures 3, 4).

**Table 3:** Characteristic findings of gallbladder polyps in CH-EUS.

	**CH-EUS findings**	**Benign**	**Malignancy**
Gallbladder polyps	Irregular intratumoral vessel	Absence	Presence
	Perfusion defect	Absence	Presence

CH-EUS, contrast enhanced harmonic endoscopic ultrasound.

### Diagnosis of gallbladder wall thickening

#### Fundamental B mode

**Figure 5:**
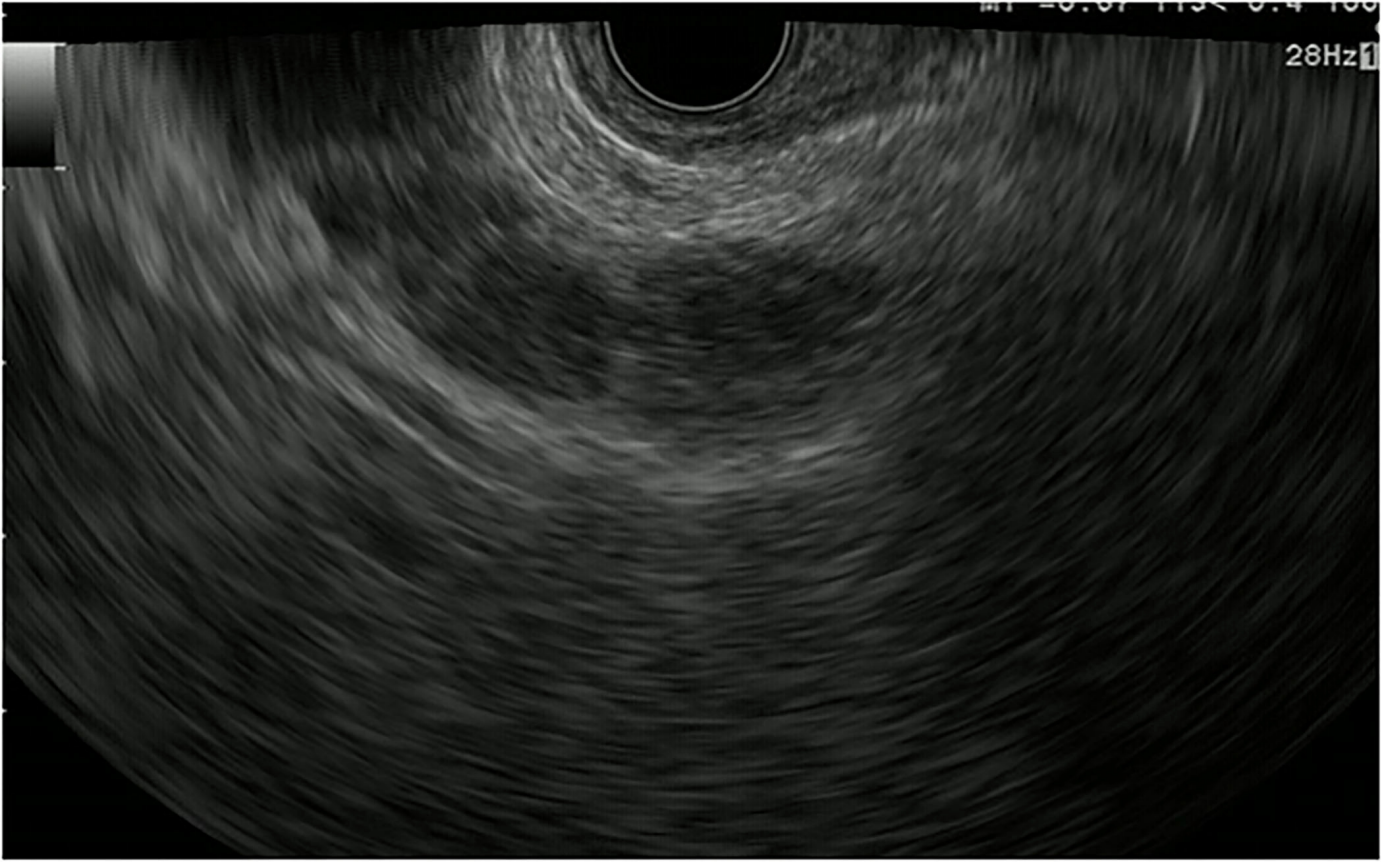
Fundamental B-mode EUS for gallbladder wall thickening. Adenomyomatosis: gallbladder wall thickening (white arrow) with a uniform surface and intramural microcystic anechoic area.

The gallbladder wall is composed of four layers: mucosa, lamina propria, muscularis propria, and serosa. Gallbladder wall thickening is defined as a gallbladder wall measuring more than 3 mm. FB-EUS can visualize the layered structure of the gallbladder and provide high-resolution images with the use of high ultrasound frequencies. Evaluation of gallbladder wall thickening is necessary for the differentiation of benign lesions such as adenomyomatosis and cholecystitis from advanced gallbladder cancer. The characteristic findings of adenomyomatosis on FB-EUS imaging are wall thickening with a uniform surface and intramural microcystic anechoic area indicating the presence of Rokitansky-Aschoff sinuses, and the “comet tail” artifact, which is a form of reverberation. However, there is a possibility of cancer coexisting with adenomyomatosis, and therefore, we have to carefully check for the presence of irregular unevenness on the surface of the adenomyomatosis during performing EUS (Figure 5).

**Figure 6:**
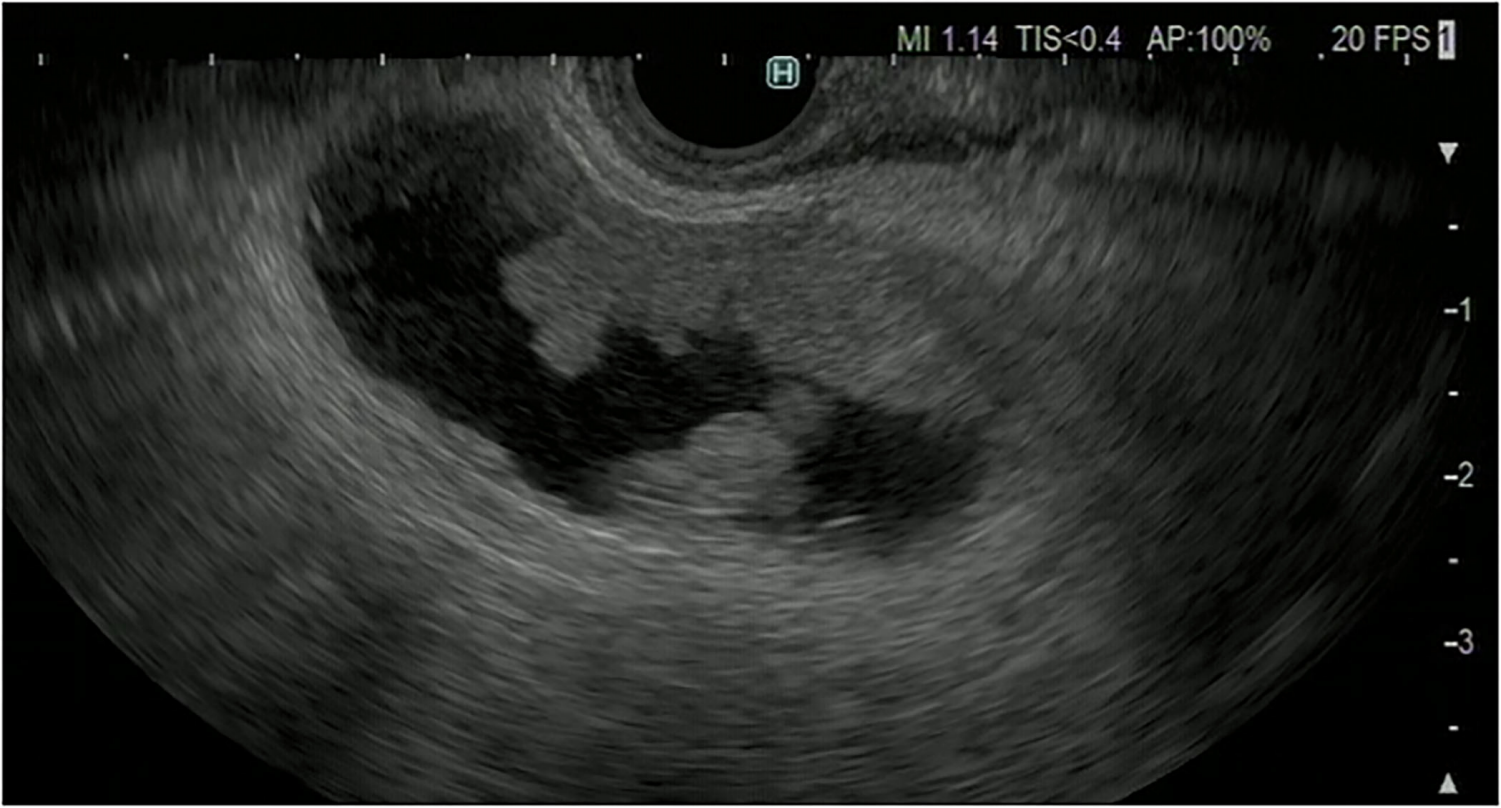
Fundamental B-mode EUS for gallbladder wall thickening. Gallbladder adenocarcinoma: gallbladder wall thickening of more than 10 mm, hypoechoic internal echogenicity, inhomogeneous internal echo pattern, and disrupted wall layer.

It is difficult to distinguish chronic cholecystitis, especially xanthogranulomatous cholecystitis (XGC), from advanced gallbladder cancer because chronic cholecystitis and XGC have a wide variety of imaging findings. XGC has findings of gallbladder wall thickening and inflammation spreading to surrounding organs, which resemble those of advanced gallbladder cancer. The characteristic findings of malignant gallbladder disease on EUS imaging include wall thickening (>10 mm), hypoechoic internal echogenicity, inhomogeneous internal echo pattern, and disrupted wall layering (21, 22) (Table 4). Kim et al. report that gallbladder wall thickening >10 mm and hypoechoic internal echogenicity are independent predictive factors for neoplastic gallbladder wall thickening (Figure 6). However, it is sometimes very difficult to diagnose gallbladder cancer using FB-EUS findings.

**Table 4:** Characteristic findings of gallbladder wall thickening in FB-EUS and CH-EUS.

	**EUS findings**	**Benign**	**Malignancy**
Gallbladder wall thickening	Wall thickening diameter	<10 mm	>10 mm
	Hypoechoic internal echogenicity	Absence	Presence
	Internal echo pattern	Homogeneous	Heterogeneous
	Gallbladder wall layer	Not disrupted	Disrupted
	Rokitansky-Aschoff sinuses	Presence (adenomyomatosis)	Absence
	Enhancement pattern on CH-EUS	Homogeneous	Heterogeneous

FB-EUS, fundamental B-mode endoscopic ultrasound; CH-EUS, contrast enhanced harmonic endoscopic ultrasound.

#### Contrast harmonic mode

**Figure 7:**
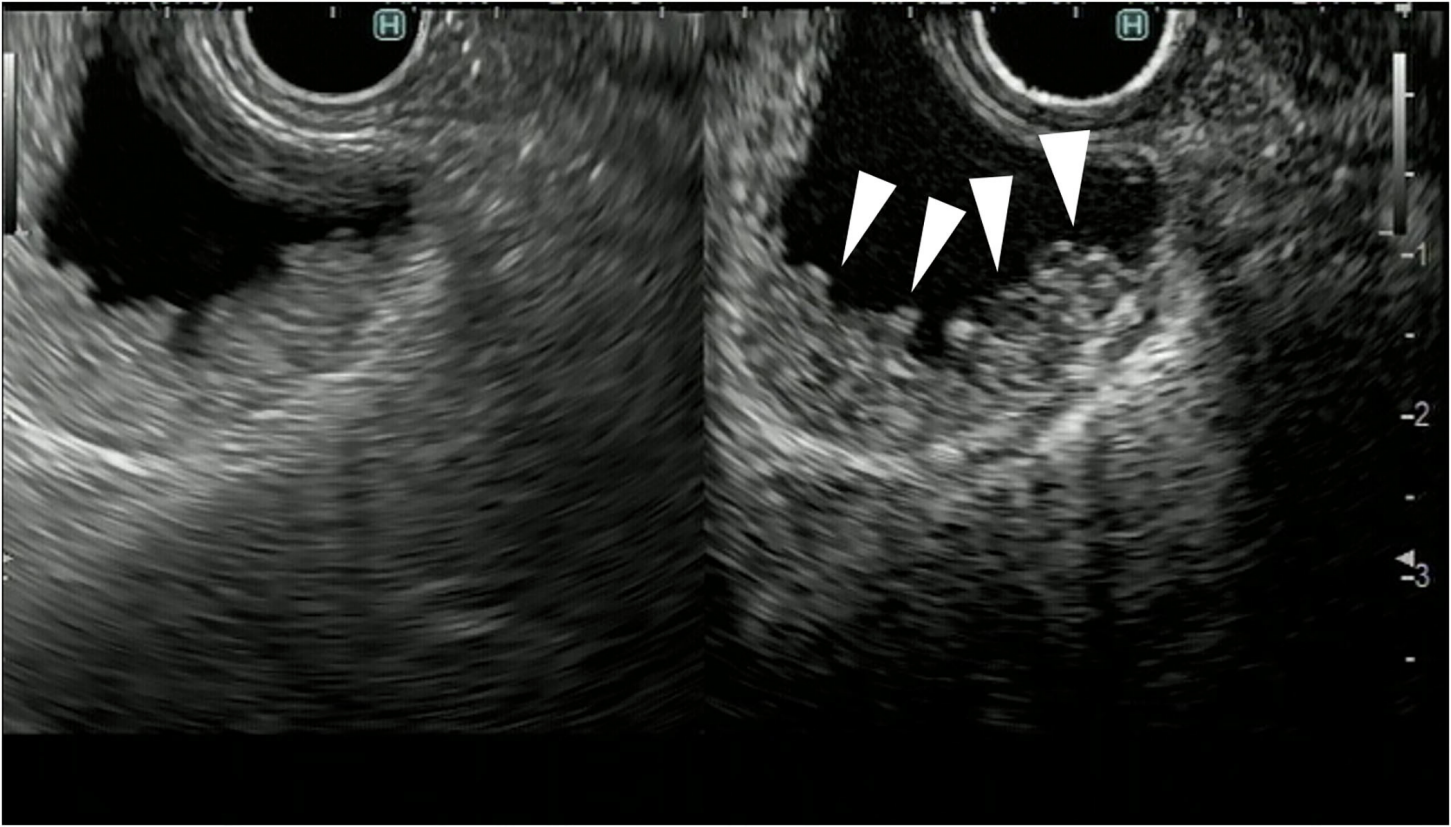
Contrast-enhanced harmonic EUS for gallbladder wall thickening gallbladder wall thickening (arrowhead) demonstrates heterogeneous enhancement.

The CH-EUS is useful for the diagnosis of gallbladder wall thickening. In gallbladder wall thickening, a heterogeneous enhancement pattern on CH-EUS is a characteristic finding of a malignant gallbladder wall (Table 4). Imazu et al. reported that CH-EUS showed significantly superior specificity and accuracy to FB-EUS in the diagnosis of malignant gallbladder wall thickening (specificity, 98 *vs*. 65%, respectively; accuracy, 94.4 *vs*. 73.1%, respectively; Figure 7) (7). When CH-EUS was added to FB-EUS, some diagnoses of cholecystitis and adenomyomatosis were changed to gallbladder carcinoma.

### Staging of gallbladder carcinoma with EUS imaging

#### Fundamental B mode

**Figure 8:**
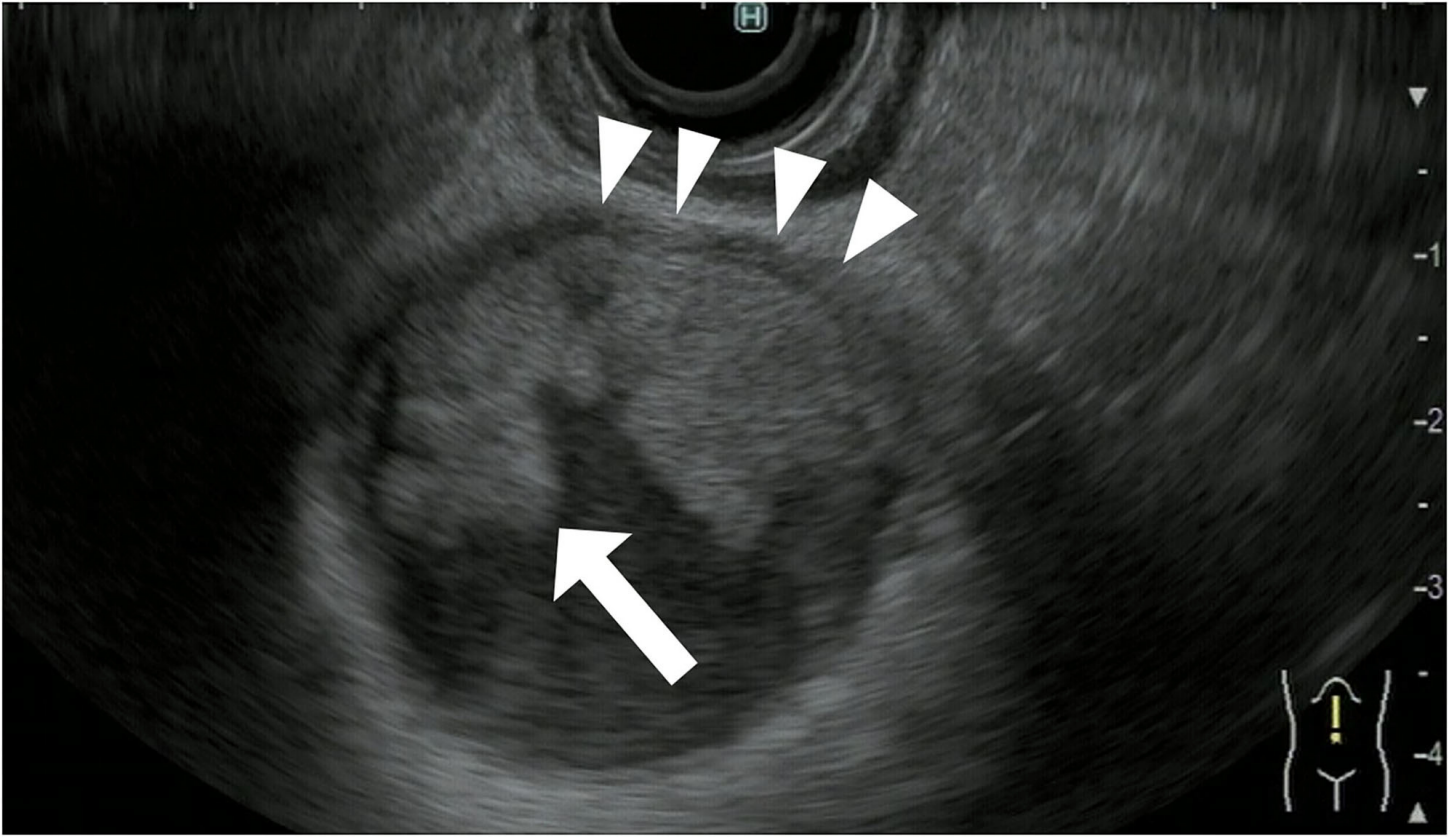
Fundamental B-mode EUS for staging of gallbladder carcinoma. T3 gallbladder carcinoma: hypoechoic tumor (arrow) in the gallbladder without a disrupted hyperechoic layer (arrowhead).

**Figure 9:**
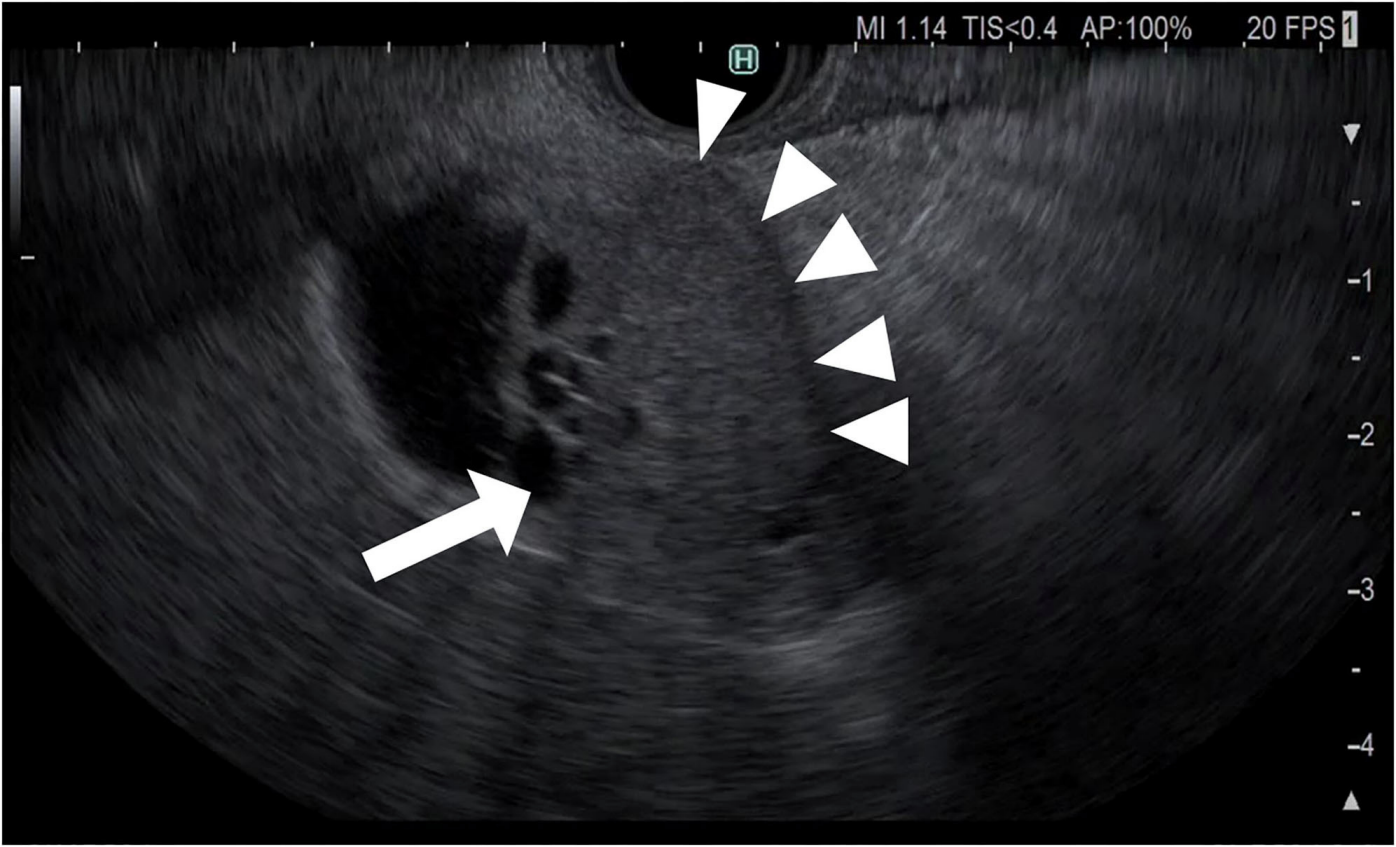
Fundamental B-mode EUS for staging of gallbladder carcinoma. T4 gallbladder carcinoma: heterogeneous hypoechoic tumor (arrow) in the gallbladder without a disrupted hyperechoic layer (arrowhead).

In the treatment of gallbladder carcinoma, it is very important to evaluate the depth of invasion of the gallbladder wall. Knowledge on whether the tumor is localized to the gallbladder or invades outside the gallbladder is very important for determining the surgical procedure for gallbladder cancer. FB-EUS is useful for evaluating the depth of invasion of the gallbladder wall because it allows clear detection of the multiple layers of the wall (23–25). As mentioned previously, the gallbladder wall is composed of four layers: mucosa, muscularis propria, subserosa, and serosa. In FB-EUS imaging, the gallbladder wall is visualized as three layers: hyperechoic, hypoechoic, and hyperechoic layers from the lumen side. The first hyperechoic layer is the boundary echo composed of mucosa and muscularis propria layers. The second hyperechoic layer is the subserosa layer (25). Assessment of this second hyperechoic layer is particularly important for determining the surgical procedure for gallbladder cancer, with cases where the second hyperechoic layer is not interrupted by the tumor being diagnosed as tumor localized to the gallbladder, and cases where this layer is interrupted being diagnosed as tumor invasion beyond the subserosa to the outside of the gallbladder (Figures 8, 9).

In addition, FB-EUS is useful for N-staging of gallbladder carcinoma. EUS is more effective to evaluate for intra-abdominal lymph node metastases than TUS. In distinguish benign and malignant lymph nodes, the most important ultrasonographic features are size, presence of a central hill, sharp, border, and cortical homogeneity (26). There are several morphological characteristics useful for distinguishing between malignant and benign lymph nodes on FB-EUS imaging. These morphological characteristics are a round sharp edge, hypoechogenicity, short axis length >13 mm, and long axis length >20 mm (27, 28). In the differentiation of malignant from benign lymph nodes, FB-EUS showed sensitivity, specificity, and accuracy of 66–81, 30–85, and 48–81%, respectively (28).

#### Contrast harmonic mode

**Figure 10:**
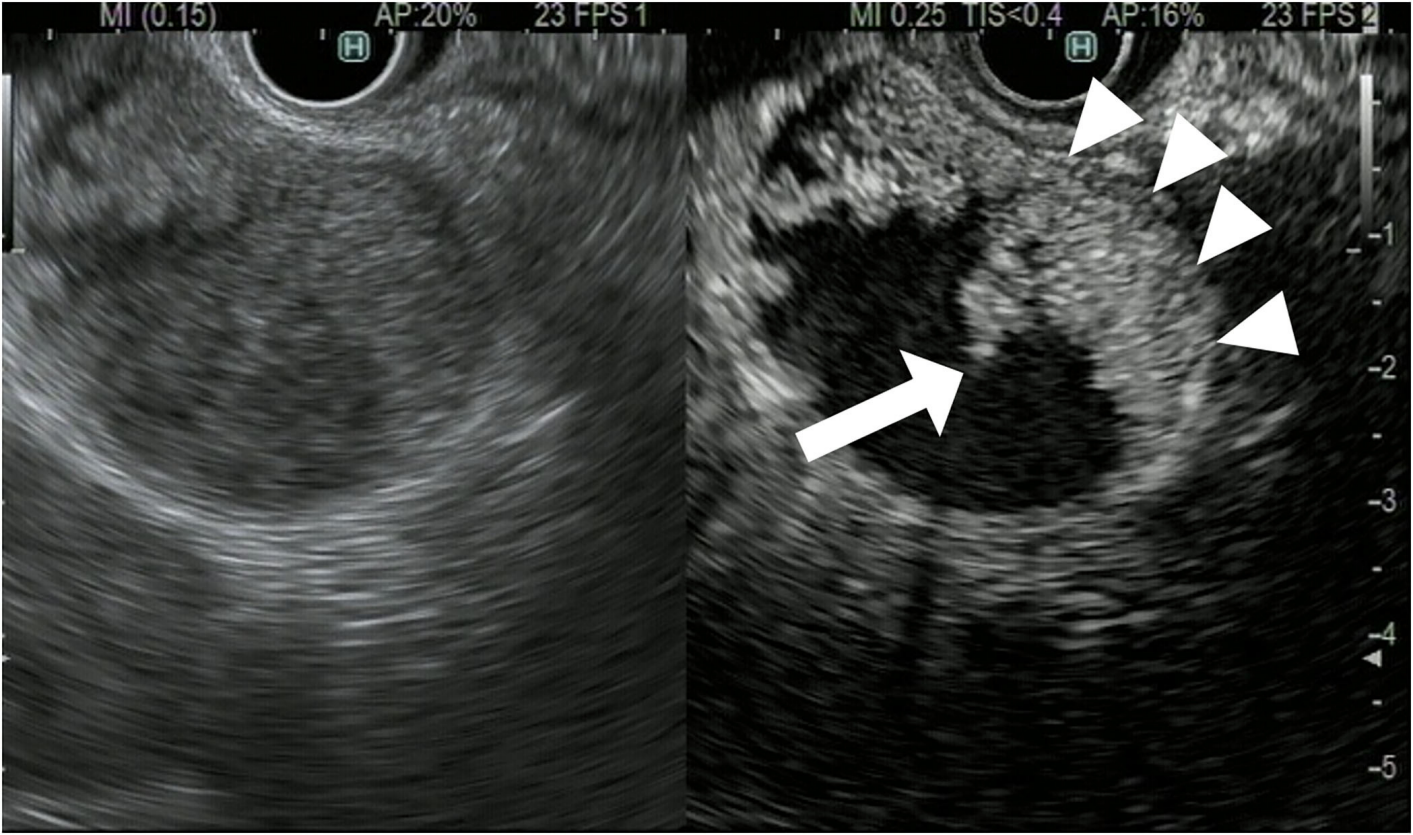
Contrast-enhanced harmonic EUS for staging of gallbladder carcinoma. T3 gallbladder carcinoma: heterogenous hyperenhancing tumor (arrow) in the gallbladder without disrupted hyperenhancement in the outer layer (arrowhead).

**Figure 11:**
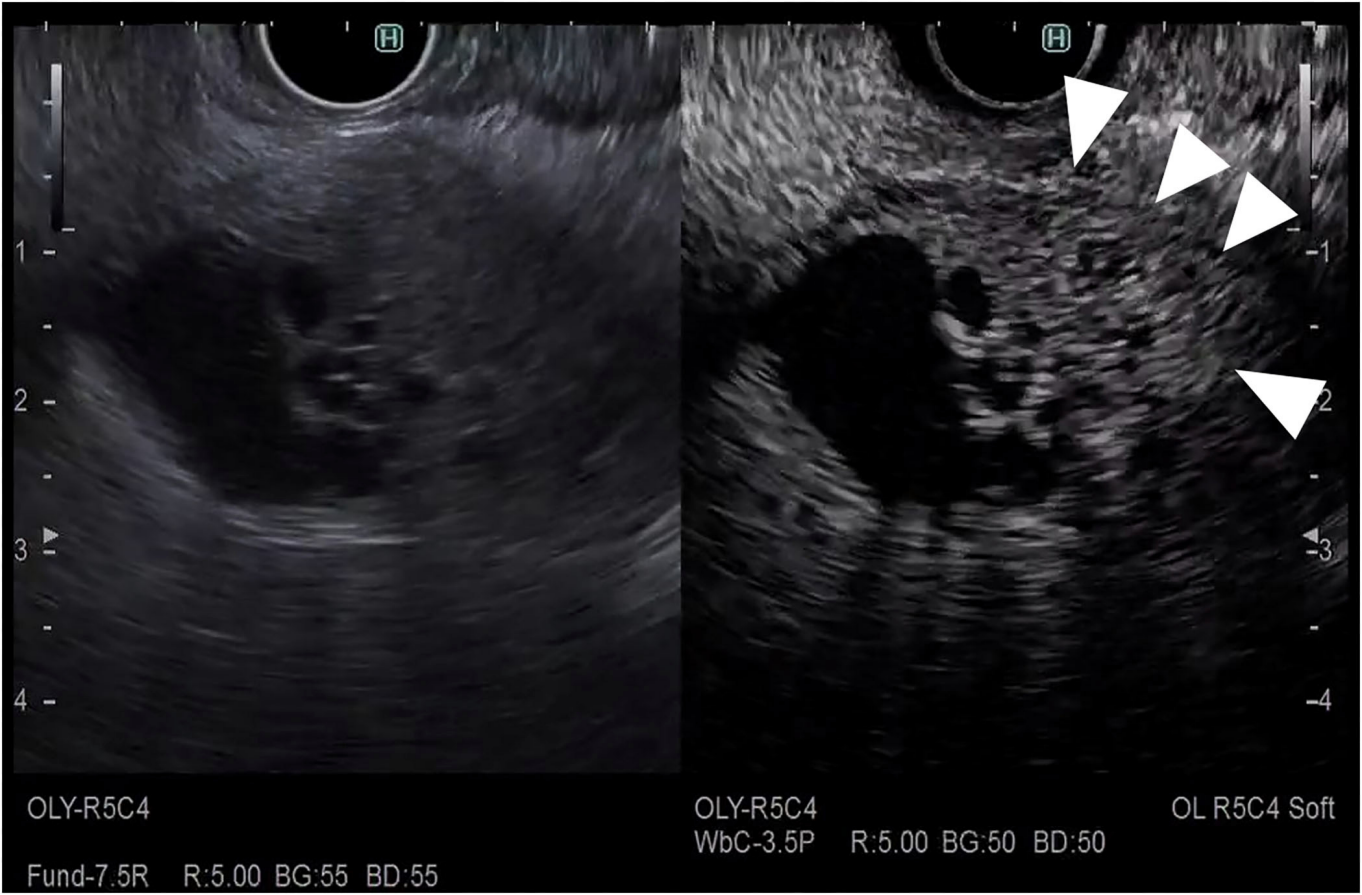
Contrast-enhanced harmonic EUS for staging of gallbladder carcinoma. T4 gallbladder carcinoma: heterogenous hypoenhancing tumor (arrow) in the gallbladder with disrupted hyperenhacement in the outer layer (arrowhead).

The CH-EUS is useful for evaluating the T-stage of gallbladder carcinoma. It was reported that CH-EUS shows the depth of invasion of biliary cancer more clearly than FB-EUS (29). On FB-EUS imaging, it can be difficult to distinguish between peritumoral inflammation tissue and hypoechoic tumors because of ultrasound attenuation due to peritumoral inflammation (30). However, on CH-EUS images, the peritumoral area with inflammation is enhanced like normal tissue, and the tissue layers between the peritumoral tissue with inflammation and the tumor are clearly visible due to the fine differences in the blood vessels in normal and tumor tissue (30–32) (Figures 10, 11).

The CH-EUS is also useful for the N-staging of gallbladder carcinoma. Differences in the enhancement pattern of CH-EUS imaging between malignant and benign lymph nodes are useful for diagnosing malignant lymph nodes (29). On CH-EUS imaging, malignant lymph nodes show a heterogeneous enhancement pattern, whereas benign lymph nodes show a homogenous enhancement pattern. When CH-EUS was used to differentiate malignant from benign lymph nodes, the sensitivity, specificity, and accuracy were 83, 91, and 88%, respectively, with the accuracy of CH-EUS in the diagnosis of malignant lymph nodes being significantly higher than that of FB-EUS (28).

## B. EUS-FNA (EUS-guided fine needle aspiration)

### Methods for EUS-FNA

The EUS-FNA puncture routes for gallbladder mass lesions include those from the duodenal bulb, gastric antecubital, and gastric body.

- We detect the gallbladder mass lesion by EUS. Next, we locate a site where we can puncture the gallbladder mass without going through the fluid space. EUS-FNA of a gallbladder mass lesion has several risks, such as bile leak and needle track seeding. Therefore, we attempt to avoid puncturing the gallbladder mass through any intervening layer of fluid or any potential space. We then targeted the mass by reorienting the probe simply by changing the position of the EUS.
- When we puncture the gallbladder wall directly with the FNA needle (guided by EUS), we have to puncture the gallbladder tumor in a tangential direction to increase the stroke distance of the moving needle. If we find that the lesion has infiltrated the liver parenchyma, or we suspect that this is the case, infiltrated liver parenchyma or the part of the gallbladder wall in contact with the liver parenchyma is an appropriate puncture site.
- After a puncture, the inner tube of the FNA needle is pulled out. Tumor cells are collected by repeatedly (10–20 times) penetrating inside the mass while applying a syringe with 20 ml of suction pressure to the FNA needle. After the needle is removed from the endoscopic channel, the stylet is reinserted and the specimen is transferred to the watch glass.
- The specimens obtained by EUS-FNA are submitted for cytology and histology. A portion of the specimen is used for ROSE. If the cytopathologist indicates that a sufficient number of cells are present, the procedure is discontinued.

### Diagnosis of gallbladder tumor with EUS-FNA

In gallbladder disease, it is necessary to obtain pathological evidence in order to determine the treatment methods. Traditionally, tissue sampling of gallbladder mass lesions has been performed by TUS, computed tomography (CT)-guided FNA, or surgery (33–35). These methods have a sensitivity of over 88% and specificity of almost 100%. However, percutaneous aspiration methods may not be able to perform in patients with small gallbladder lesions (9, 33–37), and have risks of abdominal pain (4.5%), bile peritonitis (1–6%), and needle tract seeding (9, 37). In recent years, EUS-FNA has been reported to be useful for obtaining sufficient tissue samples from various organs (38), such as gallbladder mass lesions (4, 5, 38–41).

In six studies including a total of 101 patients with gallbladder lesions, EUS-FNA showed a sensitivity of 91.7% and specificity of 100% (4, 5, 38–41). EUS-FNA is particularly useful in the diagnosis of XGC, which is difficult to distinguish from gallbladder cancer on imaging (11–14). To date, no significant adverse events (such as bleeding, bile peritonitis, and needle track seeding) of EUS-FNA have been reported. However, most studies included low numbers of patients, and all were performed retrospectively. Therefore, EUS-FNA of gallbladder tumor is rarely performed to obtain a histological diagnosis because the procedure carries the risk of bile leak and needle track seeding. In EUS-FNA for pancreatic cancer, CH-EUS guidance for FNA is reportedly useful for obtaining pancreatic tissue (42). In addition, CH-EUS guidance for FNA is reported to be useful for avoiding gallbladder complications such as bile peritonitis and needle track seeding. In cases in which it is difficult to distinguish gallbladder tumor from adjacent sludge on FB-EUS, CH-EUS allows the clear discrimination of sludge from gallbladder tumor and gallbladder wall. Therefore, under CH-EUS guidance, gallbladder tumors can be easily punctured while avoiding the puncturing of fluid spaces (43, 44).

### Indications for EUS-FNA of gallbladder tumors

There is no clear indication for EUS-FNA in the diagnosis of gallbladder tumors, although two studies have investigated the potential indications. In one study, 50 patients with gallbladder tumors underwent EUS-FNA (10). In the case of gallbladder tumor with liver and/or lymph node metastases, the metastases should be punctured first before the gallbladder mass lesion is punctured, because puncturing the gallbladder mass lesion carries the risk of bile duct peritonitis and needle track seeding. EUS-FNA was found to be more sensitive than endoscopic retrograde cholangiography sampling in gallbladder carcinoma (96 *vs*. 47.4%, *p* < 0.001) (10). In a study on the diagnosis of gallbladder tumors, gallbladder tumors were directly punctured in ten patients, lymph nodes were punctured in 37 patients, and metastatic liver lesions were punctured in two patients (4). EUS-FNA was performed in 101 patients with gallbladder mass lesions with biliary obstruction in a second large clinical trial (41). Gallbladder tumors were punctured in 58 patients, lymph nodes in 23, and both in 16. The sensitivity and specificity of EUS-FNA for gallbladder tumor were 90.8 and 100%, respectively. There were no serious adverse events caused by EUS-FNA (41).

On the basis of these studies, the following strategy for EUS-FNA for gallbladder tumor is recommended.

- In patients with gallbladder tumors with liver and/or lymph node metastases, the liver and/or lymph node metastases should be punctured before the gallbladder tumor is punctured.
- In EUS-FNA for gallbladder tumor, puncturing of the gallbladder mass lesion is preferably indicated for large mass lesions such as those spreading in the biliary duct or infiltrating the liver because it is easy to puncture the gallbladder mass lesion while avoiding the lumen of the gallbladder.
- In patients with gallbladder tumor localized to the gallbladder, endoscopic transpapillary gallbladder drainage (ETGBD) can be used to evaluate the cytopathology of tumors, although ETGBD has drawbacks such as insufficient diagnostic accuracy and the need for a specialized endoscopist. In the case of failed ETGBD, EUS-FNA is an alternative option for the pathological diagnosis of gallbladder tumors.

### Staging of gallbladder carcinoma with EUS-FNA

The staging of gallbladder carcinoma is important for determining treatment. In particular, the presence of distant metastasis and para-aortic lymph node (PALN) metastasis are important factors for determining whether or not surgery is possible. PALN metastasis is classified as distant metastasis and is regarded as an unresectable factor according to the Union for International Cancer Control (UICC) (45). ^18^F-Fluorodexyglucose positron emission tomography (FDG-PET) and FDG-PET with CT (PET/CT) are often used for the diagnosis of lymph node metastasis. However, the accuracy rate for diagnosis of lymph node metastasis on PET/CT is only 60–80%, which is not satisfactorily high (46–48). In some studies, EUS-FNA was reported to be useful for the diagnosis of lymph node and PALN metastasis (48–50). Kurita et al. reported that EUS-FNA had higher sensitivity and specificity (96.7 and 100%, respectively) than PET/CT in the diagnosis of PALN metastasis (50). It was also reported that EUS-FNA had higher sensitivity and specificity than EUS in the diagnosis of PALN metastasis. EUS-FNA was shown to be superior to PET/CT for preoperative PALN staging in patients with gallbladder carcinoma (48).

### Genetic analysis of gallbladder tumor with EUS-FNA

Recently, with the development of next-generation sequencers, the number of patients undergoing individualized medicine based on genome biomarkers is increasing. Gallbladder and biliary tract cancers have driver genes such as ERBB2, PIK3CA, IDH1/2, BRCA1/2, and FGFR2 fusion genes (51–53). It was reported that next-generation sequencing is possible for gallbladder cancer tissue obtained by EUS-FNA. Therefore, in the future, EUS-FNA may become an even more essential examination when deciding on chemotherapy for gallbladder cancer (54).

## Conclusion

The FB-EUS and CH-EUS are very useful examinations for differentiating between benign and malignant gallbladder tumors and the staging of gallbladder carcinoma. EUS-FNA is not only a useful examination in the diagnosis and staging of gallbladder carcinoma, but is also becoming an essential examination for determining the choice of chemotherapy.

## Author contributions

TT and MK manuscript writing, drafting conception, and design. RA critical revision of the article for important intellectual content. All authors read and approved the final version of the manuscript.

## Conflict of interest

The authors declare that the research was conducted in the absence of any commercial or financial relationships that could be construed as a potential conflict of interest.

## Publisher's note

All claims expressed in this article are solely those of the authors and do not necessarily represent those of their affiliated organizations, or those of the publisher, the editors and the reviewers. Any product that may be evaluated in this article, or claim that may be made by its manufacturer, is not guaranteed or endorsed by the publisher.
